# MOGAT2: A New Therapeutic Target for Metabolic Syndrome

**DOI:** 10.3390/diseases3030176

**Published:** 2015-08-28

**Authors:** Muhua Yang, Joseph T. Nickels

**Affiliations:** Institute of Metabolic Disorders, Genesis Biotechnology Group, Hamilton, NJ 08691, USA

**Keywords:** triglyceride, metabolic disorders, obesity, acyltransferases

## Abstract

Metabolic syndrome is an ever-increasing health problem among the world’s population. It is a group of intertwined maladies that includes obesity, hypertriglyceridemia, hypertension, nonalcoholic fatty liver disease (NAFLD), and diabetes mellitus type II (T2D). There is a direct correlation between high triacylglycerol (triglyceride; TAG) level and severity of metabolic syndrome. Thus, controlling the synthesis of TAG will have a great impact on overall systemic lipid metabolism and thus metabolic syndrome progression. The Acyl-CoA: monoacylglycerolacyltransferase (MGAT) family has three members (MGAT1, -2, and -3) that catalyze the first step in TAG production, conversion of monoacylglycerol (MAG) to diacylglycerol (DAG). TAG is then directly synthesized from DAG by a Acyl-CoA: diacylglycerolacyltransferase (DGAT). The conversion of MAG → DAG → TAG is the major pathway for the production of TAG in the small intestine, and produces TAG to a lesser extent in the liver. Transgenic and pharmacological studies in mice have demonstrated the beneficial effects of MGAT inhibition as a therapy for treating several metabolic diseases, including obesity, insulin resistance, T2D, and NAFLD. In this review, the significance of several properties of MGAT physiology, including tissue expression pattern and its relationship to overall TAG metabolism, enzymatic biochemical properties and their effects on drug discovery, and finally what is the current knowledge about MGAT small molecule inhibitors and their efficacy will be discussed. Overall, this review highlights the therapeutic potential of inhibiting MGAT for lowering TAG synthesis and whether this avenue of drug discovery warrants further clinical investigation.

## 1. Introduction

TAG stores and transports fatty acids (FAs) as energy for fuel and provides substrates for lipid synthesis that maintain membrane phospholipid synthesis [1]. In mammals, adipose tissue stores TAG as a primary energy substrate to sustain animals during fasting. TAG also protects cells from lipotoxicity, provides natural ligands for nuclear hormone receptors, and is involved in cell signaling [2]. A hallmark of a variety of metabolic pathological conditions including obesity, insulin resistance, T2D, coronary heart disease, hypertriglyceridemia, and NAFLD is the excessive accumulation of TAG in human adipose and non-adipose tissue [3,4]. TAG-rich lipoproteins comprise a vast array of secreted particles from intestine and liver. Multiple clinical trials and models have established the relationship between elevated plasma TAG and increased risk of coronary and cerebrovascular ischemic events [5]. Mendelian randomization studies have also demonstrated a causal involvement of TAG-mediated pathways and conronary heat disease [6]. Limiting TAG production in humans provides a potential pharmacological intervention of these metabolic disorders.

### 1.1. TAG Biosynthesis

**Figure 1:**
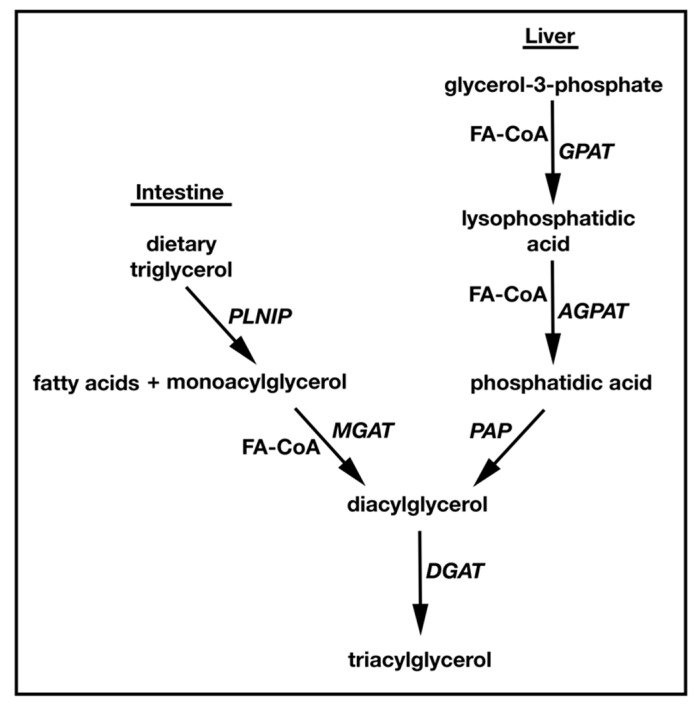
Schematic representation of the two triglyceride synthesis pathways using either MAG or G3P as the initial acyl acceptor. The G3P pathway is dominant in liver, whereas MGAT mediated MAG to TAG conversion is predominant in the intestine.

TAG synthesis and metabolism have been topics of study since the 1950s. The conserved pathway for TAG synthesis in all higher eukaryotes is the Kennedy or sn-glycerol-3-phosphate (G3P) pathway [7] (Figure 1). Here, G3P is acylated by glycerol-3-phosphate acyltransferase (GPAT) to produce lysophosphatidic acid (LPA). LPA is then acylated by acylglycerol-3-phosphate acyltransferase (AGPAT) to generate phosphatidic acid (PA), which is dephosphorylated by lipins/PA phosphatases (PAP) to generate DAG [8,9,10,11,12]. Finally, DAG is converted to TAG by DGAT. TAG can also be synthesized by a second distinct MAG-dependent pathway that is found exclusively in animals, playing a predominant role in dietary fat absorption in the small intestine [13,14]. Here, MAG is converted to DAG by MGAT enzymes (MGAT1/2/3), and like the G3P pathway, DAG is converted to TAG by DGAT. While the G3P pathway uses G3P as an initial acyl acceptor, the MAG pathway uses MAG. Interestingly, both pathways use acyl-CoA thioesters as acyl donors to produce DAG, and both converge at the final DGAT acylation step to produce TAG [15,16,17,18] (Figure 1). The endoplasmic reticulum (ER) is the main site for these reactions, but mitochondria can also synthesize TAG. Interestingly, lipid droplets can produce the TAG precursors LPA and PA [1,19] and it has been shown that dihydroxyacetone phosphate (DHAP) can be esterified to form the LPA precursor, 1-acyldihydroxyacetone-phosphate in all animal tissues and yeast [20,21].

TAG is *de novo* synthesized in the liver and adipose tissue, while dietary TAG is broken down and re-synthesized in the small intestine. In the liver, TAG is used for very low density lipoprotein (VLDL) assembly. Newly formed VLDL is secreted into the circulatory system where it transports neutral lipids including TAG to peripheral tissues [1]. In the small intestine, dietary TAG is hydrolyzed by pancreatic lipase to FA and MAG that are re-absorbed in the intestinal lumen. Enterocytes then re-synthesize TAG and secrete it as ApoB-containing chylomicrons that deliver dietary fat to tissues [22]. Most tissues including liver and adipose use the G3P pathway for the synthesis of TAG. In contrast, the small intestine predominately relies on the MAG pathway [23,24,25,26].

As stated above, MGAT activity is essential for the biosynthesis of TAG in the small intestine. The fact that MGAT expression level is found to be elevated in humans with obesity, while hepatic MGAT expression is decreased in patients after Reux-en-Y gastric bypass (RYBG) surgery [27,28] indicates that MGAT itself is a major regulator of TAG homeostasis in response to diet. Below, the structural and functional aspects of the MGAT family of proteins (MGAT1, MGAT2 and MGAT3) will be discussed. Related acyltransferases (e.g., DGAT1) will be touched upon as to their molecular relationship with MGAT. Finally, the review will examine whether MGAT is a viable pharmacological target for the treatment of metabolic disorders.

## 2. Identification and Characterization of MGATs

Monoacylglycerolacyltransferase (MGAT) is an ER bound enzyme [29,30,31].Three known MGAT genes, Mogat1, Mogat2 and Mogat3, were identified in the early 2000s based on their sequence homology with the DGAT2 gene [29,30,31,32]. In mammals, MGAT is involved in intestinal dietary fat absorption and TAG synthesis in suckling rat liver, as very little MGAT activity has been detected in adult rat liver microsomes [33]. Interestingly, MGAT genes share homology with DGAT2 but not DGAT1.

### 2.1. MGAT1

MGAT1 was the first family member to be cloned from mouse liver in 2002 [31] based on sequence homology to DGAT2 [32]. Mouse MGAT1 is located on chromosome 1, while in humans it localizes to chromosome 2. MGAT1 contains a core amino acid sequence that shares homology to a domain found in phosphate acyltransferases, two putative N-linked glycosylation sites, and a possible tyrosine phosphorylation site. Interestingly, MGAT1 does have *in vitro* DGAT activity, as demonstrated using a radio-labeled DGAT activity assay. However, activity towards DAG is significantly less than DGAT2 [31]. Mouse MGAT1 expression is found to be highest in the stomach and kidney; it is also present at a lower level in white and brown adipose tissue, uterus, and liver. Importantly, MGAT1 expression is found to be absent in mouse small intestine.

Human MGAT1 is also expressed in the stomach, uterus, kidney, adipose, and liver, but unlike the mouse, expression is seen in brain, lung, thymus, prostate, testes, colon, and notably, small intestine [31]. There are two identified splice variants of human MGAT1, the larger of the two is predominantly found in the thymus and testes [34]. Work from Hall *et al.*, showed that the putative full-length MGAT1 transcript was not amplified in human liver, gall bladder, adipose, or intestine, which suggests the presence of multiple splicing variants [27].

The function of MGAT1 outside the small intestine has yet to be elucidated. One possibility is that it plays a role in polyunsaturated FA preservation in tissues other than the small intestine, where fast turnover of TAG degradation and re-synthesis occurs [33]. For example, when FA is required as the major source of energy in suckling rat and chick embryo, despite the high rate of hepatic beta-oxidation, the long-chain polyunsaturated derivatives of FAs (e.g., C18:2n-6 and C18:3n-3) are selectively retained. The preference of MGAT activity for MAGs that contain C18:2 and C18:3 FA in the sn-2 position could lead to greater retention of essential FAs by preventing them from being used during β-oxidation [33]. In humans, the expression of MGATs is up-regulated in the livers of insulin-resistant patients who have nonalcoholic fatty liver disease (NAFLD) [27]. In the mouse, MGAT1 (the dominant isoform) expression and activity are increased in diet-induced obese (DIO) and ob/ob mice. This up regulation may be explained by increased expression of the hepatic peroxisome proliferator-activated receptor (PPAR)γ, which directly regulates MGAT1 promoter activity [35].

Antisense oligonucleotide (ASO) knockdown of MGAT1 in mice fed a high trans FA, fructose, and cholesterol diet (HTF-C diet) prevented weight gain, improved glucose tolerance and hepatic insulin signaling, and decreased hepatic TAG content when compared to control ASO-treated mice fed the same diet (Table 1) [36]. Similar findings were observed for DIO and ob/ob MGAT1 ASO treated mice [37]. Interestingly, in spite of the metabolic beneficial effects due to knockdown of MGAT1, no reduction was seen in hepatocyte ballooning, macrophage infiltration, or stellate cell activation, nor did it attenuate the expression of inflammation markers [36]. Unexpectedly, the knockdown of MGAT1 increased total DAG content in both membrane and cytosolic compartments of hepatocytes [37]. The increased DAG accumulation was not due to compensatory increases in phosphatidic acid phosphatase or TAG hydrolase activities, but may have been due to decreased DGAT activity; as stated above MGAT1 does have DGAT activity [31]. Thus, although reducing intrahepatic lipid content will most likely protect the liver from the development of nonalcoholic steatohepatitis (NASH), inhibition of TAG synthesis by MGAT1 inhibition may not necessarily be beneficial, at least in mice. Important to this statement is the fact that MGAT1 protein expression is undetectable in humans. Thus, small molecule discovery targeting MGAT2 should not have to be concerned with potential cross-reactivity, selectivity issues, and toxicity associated with inhibiting MGAT1.

### 2.2. MGAT2

The first mammalian MGAT2 was cloned and characterized in mice in 2003 [13], with cloning of the human MGAT2 coming shortly afterwards [30,38]. Human MGAT2 shares 81% identity with the mouse MGAT2 and 47% amino acid identity with DGAT2. MGAT2 and DGAT2 localize adjacent on chromosome 11q13.5, suggesting a possible duplication event. Human MGAT2 has a splice variant (hMGAT2V) that lacks MGAT2 activity.

Human MGAT2 is primarily expressed in the small intestine and liver. It can also be detected in adipose and colon at a much lower expression level. hMGAT2V also shows expression in the small intestine, but what its molecular function is remains to be elucidated. MGAT2 is predominantly expressed in the small intestine and kidney in the mouse [13]. While MGAT2 is found in human liver, its activity is barely detectable in human liver microsomes. This finding was in line with reports that showed hepatic MGAT2 specific activity was very low in adult rat, chick, and guinea pig compared with neonatal rat, fetal guinea pig, and chick embryo [33,39,40,41]. Thus, hepatic TAG synthesis in adult mammals is thought to occur primarily via the sequential acylation of G3P. The lack of detectable MGAT activity in human liver may be due to the very high lipase activity in adult liver, which depletes the MAG substrate required for DAG synthesis by MGAT2 [42]. This is backed up by experiments using the lipase inhibitor, MAFP, which showed there was active MGAT2 activity in human liver microsomes [27]. There is still a debate whether this activity truly comes from MGAT2 or MGAT3, as MGAT2 protein cannot be detected in human liver microsomes [27]. Taken together, the majority of the present data indicates that MGAT2 activity is substantially lower in human liver than in the small intestine.

**Table 1:** Genetic Manipulation of MGAT1 and MGAT2 in Mouse models.

Genetic Manipulation	Mouse Model	Description	TAG Content		Reference
			Plasma	Liver	
Mogat1 ASO	DIO	Weight loss was seen in all but DIO model;	n.c.	n.c.	[35,36,37]
	ob/ob	Fatty acid oxidation was increased; Lipogenesis was suppressed;	↓	n.c.	
	HFD	Insulin sensitivity was improved; Hepatic inflammation was enhanced.	n.c.	↓	
Mogat2 ^−/−^ Mogat2^iko^	HFD	Weight and food intake was decreased; Food preference was changed to carbohydrate from fat; Plasma non-HDL cholesterol was decreased; Hepatic steatosis was prevented; Energy expenditure was increased; GLP-1 secretion was increased; Insulin sensitivity was improved.	↓	n.d.	[43,44]

n.c., no change; DIO, diet-induced obesity; HFD, high fat diet; n.d., not determined.

Much information has been gleaned about MGAT2 function using transgenic mouse models (Table 1). Mogat2^−/−^ mice are metabolically healthy, are resistant to high-fat diet induced obesity, have improved insulin sensitivity, and have decreased fat accumulation in the liver and adipose tissue [43]. Moreover, the deletion of MGAT2 increases the level of gut incretin glucagon-like peptide-1 (GLP-1), which has many beneficial effects in treating metabolic disorders including stimulating insulin secretion, reducing glucagon, slowing gastric emptying, and inducing satiety [45]. Interestingly, Mogat2^−/−^ mice fed a high fat diet consume and absorb a similarly high percentage of dietary fat when compared to their wild-type litter mates on the same diet. The fat level in the feces of Mogat2^−/−^ mice was not increased and vitamin A and E levels were normal, indicating MGAT2 is dispensable for fat absorption. Both whole body Mogat2^−/−^ and intestine-specific deletion (Mogat2^iko^) mice exhibit significantly lower intestinal MGAT2 activity and have impaired intestinal TAG synthesis [44]. MGAT2 deficiency also slows gastric emptying and changes the kinetics of fat absorption.

Mogat2^−/−^ mice display an increase in energy expenditure when compared to their wild-type counterparts. Re-introduction of MGAT2 in the intestine of Mogat2^−/−^ mice remediates the MAG absorption defect, thus allowing for the conversion of dietary MAG → DAG → TAG, and restores the rate to which dietary fat enters the circulation [46]. Mogat2^iko^ mice also show an energy expenditure rate that is somewhere in between Mogat2^−/−^ and wild-type mice [46]. Data from Nelson *et al.*, indicated that the change in energy expenditure in Mogat2^−/−^ mice was unrelated to high-fat diet consumption, as when these mice were fed a normal chow diet increased energy expenditure remained intact [47]. In contrast, data from Mul *et al.*, showed that Mogat2^−/−^ mice only increased their energy expenditure when fed a high-fat diet but not with normal chow [28]. Interestingly, Mogat2^−/−^mice have a preference for eating carbohydrate over fat, which correlates with an increase in locomotive activity, suggesting that shifting macronutrient preference away from fat may act as a protective mechanism against high-fat diet induced obesity [28]. Thus, targeting MGAT2 for inhibition may have beneficial effects on obesity.

Adult-onset MGAT2 deficiency in mice brought on by expressing a tamoxifen-inducible Cre recombinase demonstrated that MGAT2 was involved in regulating energy balance. Somatic loss also protected mice against diet-induced weight gain, hepatic steatosis, and glucose intolerance. Just as important is the fact that inactivation of MGAT2 in diet-induced obese mice reduced their body weight and improved glucose tolerance [48]. This observation indicates that MGAT2 deficiency during early development is not required for the protective effects MGAT2 inactivation gives. However, germ line MGAT2 knockout mice do suffer from malnutrition and gain less weight during the suckling postnatal period. Moreover, the reduction in weight gain in MGAT2-inactivated adult mice is less than germ line MGAT2 knockouts. With that said, these results do indicate that targeting MGAT2 for small molecule discovery will be efficacious, and that adult patients with various metabolic disorders will respond to treatment.

The accumulation of specific lipid intermediates, including DAG, acyl-CoA, and ceramide is thought to drive the progression of NAFLD in humans [49,50]. Although in the liver, most DAG and TAG are synthesized through the sequential acylation of G3P. MGAT2 may provide an alternative pathway for the generation of DAG. Unlike in mice, human MGAT2 mRNA expression can be detected in liver, as well as in small intestine [27,34]. While MGAT2 mRNA is highly expressed in pooled human liver RNA, MGAT2 protein expression is not detectable in human liver lysates among 200 pooled donor samples. In contrast, pooled human intestine samples have robust MGAT2 protein expression. This may suggest that liver MGAT2 expression may be under tight promoter regulation, or that the stability of MGAT2 protein is highly regulated due to the detrimental consequences of liver TAG accumulation [27]. Thus, the question of why the MGAT2 pathway is found in human liver still needs to be investigated. As stated above, and importantly, MGAT2 expression is up regulated in patients with obesity and NAFLD, and down regulated following gastric bypass surgery. This suggests that MGAT2 may have a positive role in lipid-related liver disease progression. As there are very few therapeutics out there to treat NAFLD, targeting of MGAT2 for inhibition may not just help in lowering TAGs, but may also potentially reduce or eliminate the progression of NAFLD, which ultimately progresses to NASH, and later cirrhosis.

### 2.3. MGAT3

Among the three MGAT isozymes identified to date, the MGAT3 gene, Mogat3, is thought to only exist in higher mammals and not in rodents, one publication has documented its expression and function in rats (discussed below); murine Mogat3 is a pseudogene. Human MGAT3 was cloned in 2003 and it has been shown that over-expressing MGAT3 in baculo virus gives robust MGAT enzyme activity. Like MGAT2, MGAT3 appears to have substrate specificity for the acylation of 2n-monoacylgycerol over other stereoisomers [51]. MGAT3 was initially thought to be strictly expressed in the human intestine, however, it was shown later on that MGAT3 was expressed in human liver [27]. The sequence of MGAT3 is more homologous to DGAT2 than to MGAT1 or MGAT2.Thus, MGAT3 exhibits significantly higher DGAT activity than MGAT1 and MGAT2 when MAGs or DAGs were used as substrates, suggesting another gene duplication event giving rise toMGAT3 from DGAT2 [29].

Besides being expressed in human intestine, MGAT3 expression has been seen in pooled human liver RNA and protein in cell lysates. MGAT3 expression level increases in patients with NAFLD and its level decreases after gastric bypass surgery-induced weight loss. The MGAT activity present in livers of obese human subjects correlated with Mogat3 gene expression, not MGAT2 [27]. This suggests MGAT3 might play a more important role than MGAT2 in obesity related hepatic insulin resistance and NAFLD progression in humans.

As the function of murine MGAT3 has yet to be determined, its true *in vivo* physiological importance in TAG biosynthesis and absorption in mammal remains a mystery. While MGAT3 was reported to be functional in the rat [14], and its expression pattern in this rodent was shown to be similar to human MGAT3, knockout studies in rodents are lacking. Until then, the functional relationship between MGAT2 and MGAT3 will remain unanswered. For instance, can MGAT3 compensate for MGAT2 function when MGAT2 is inhibited? What is the substrate specificity of MGAT3, and how does that affect lipid metabolism? What is the role of MGAT3 in NAFLD progression? There are a number of excellent hypotheses that can be tested once transgenic animals are created.

### 2.4. DGATs

As discussed above, MGAT isoforms share sequence homology and similar substrate specificities with the DGAT family member, DGAT2. Two DGAT isozymes have been identified in mammals: DGAT1 and DGAT2 [32,52]. The DGATs do not share similar tissue expression patterns (DGAT1 is mainly expressed in adipose and small intestine; DGAT2 is expressed in liver). Moreover, DGAT1 does not share sequence homology with DGAT2, again suggesting duplicate functions between DGAT2 and MGATs. DGAT2 knock mice are lipopenic and die shortly after birth [53] due to profound reductions in substrates for energy metabolism and an impaired permeability barrier in the skin. Interestingly, DGAT1 expression was unable to compensate for the loss of DGAT2, indicating a fundamental role for DGAT2 in TAG biosynthesis, much more so than DGAT1. DGAT1 knockout mice were found to be viable and were protected from high-fat diet induced obesity and insulin resistance. For information concerning the biochemical function and physiological significance of each DGAT, the reader is referred to an excellent review of this material [54].

Pharmacological interventions using DGAT1inhibitors have been developed as a therapy for obesity and T2D. These inhibitors have been tested in rodent and dog models and have been shown to delay gastric emptying and lower fat absorption, decrease postprandial plasma TAG, increase GLP-1 level, stimulate FA oxidation, reduce liver TAG, and improve insulin sensitivity while causing weight loss [55,56,57,58,59,60,61]. In human clinical trials, similar results were seen: a reduction in postprandial plasma TAG, an increase GLP-1 level, normalized insulin sensitivity, and weight loss. However, the beneficial effects of these inhibitors are often counterbalanced by intolerable gastrointestinal side effects including nausea, diarrhea, and vomiting. These adverse symptoms are thought to be the result of on target side effects; as unlike mice, humans appear to solely rely on DGAT1 activity for TAG synthesis in the small intestine, while DGAT2 can compensate for the loss of function of DGAT1 in DGAT1 deficient (Dgat1-/-) mice. The inhibition of human intestinal DGAT enzyme blocks TAG synthesis completely and has led to severe fat malabsorption, as observed in patients with a null mutation in the DGAT1 gene [62,63]. Thus, all gastrointestinal side effects were thought to be due to the severe malnutrition and buildup of DGAT substrates. For all these reasons, the use of DGAT1 inhibitors as anti-diabetes and/or anti-obesity agents remains uncertain. Currently, there are ongoing clinical studies using DGAT1 inhibitors (Novartis) to treat patients with familiar chylomicronemia [64].

## 3. Small Molecule MGAT2 Inhibitors

The biosynthetic pathways leading to TAG synthesis are still attractive targets for pharmacological intervention even in light of the side effects associated with DGAT inhibition. One possible problem that was not foreseen in inhibiting DGAT was that both pathways converge on the DGAT step for TAG synthesis. A more attractive target may be MGAT2. The thought is that MGAT2 inhibition in the small intestines is unlikely to have DGAT-like side effects and cause severe deficiency in TAG synthesis, as the alternative G3P pathway is active in human intestine. Because of the similar expression and function of MGAT2 in human and mice, MGAT2-knockout mice have been very useful in helping in the discovery of small molecule MGAT2 inhibitors aimed at treating several lipid-related maladies, such as obesity, hypertriglyceridemia, and T2D. Another advantage of targeting MGAT2 is that drug discovery is still in the infancy stage, as there are only 10 MGAT2 inhibitor patents published. Examples of small molecule MGAT2 inhibitors are listed in Table 2.

**Table 2:** MGAT2 inhibitors and structures.

Company	Description	Reference
**Banyu**	Pyrimidine-4(3H)-one derivatives, IC_50_ = 56 nM, Selectivity to other acyltransferasesare unknown	WO2010/095767
**Dainippon Sumitomo**	Bicyclic pyrimidine derivative, IC_50_ = 2 nM, Selectivity to other acyltransferasesare unknown	WO2012091010 A1
**Taisho**	N-containing heterocyclic derivatives, IC_50_ = 4.1 nM, Selectivity to other acyltransferasesare unknown	WO2012124744 A1
**Eli Lily**	Phenyl methanesulfonamide derivatives, IC_50_ = 12 nM *in vitro* assay, IC_50_ = 17.7 nM CaCo2 cell based LC-MS assay, Selectivity to other acyltransferasesare unknown Benzyl sulfonamide derivatives, IC_50_ = 2.28 nM *in vitro* assay, IC_50_ = 3.8 nM CaCo2 cell based LC-MS assay, >70% reduction in TAG excursion in a dog oil bolus model, Selectivity to other acyltransferasesare unknown Morpholynyl derivatives, IC_50_ = 12 nM *in vitro* assay, IC_50_ = 16 nM CaCo2 cell based LC-MS assay, 43% to 64% reduction of TAG excursion in a dog oil bolus model, Selectivity to other acyltransferasesare unknown	WO 2013112323 A1 WO2014074365A1 US20150005305
**Bristol-Myers Squibb**	Aryl dihydropyridinones and piperidinones derivatives, IC_50_ = 14 nM *in vitro* assay, IC_50_ = 4 nM in STC-1 LC/MS assay, >1000-fold selective over other acyltransferases	WO2013/082345 [65]
**AstraZeneca**	3-ethyl-3-methyl-2,5-dioxo- *N*-phenyl-2,3,4,5-tetra hydro-1H-1,4-benzodiazepine-7-sulphonamide 1, IC_50_ = 1.6 nMRapidfire LCMS^®^ assay, 68% reduction of TAG excursion in a mice oil oral gavage model, >10,000-fold selective over AWAT, variable selectivity toward MGAT2 and no selectivity toward MGAT1	[66]
**Takeda**	*N*-phenylindoline-5-sulfonamide derivatives, IC_50_ = 3.4 nMRapidfire LCMS^®^ assay, Significant reduction of TAG excursion in a mice olive oil oral gavage model, >30,000 fold selective over MGAT3, DGAT1, DGAT2 and ACAT1	[67]
**Japan Tobacco Inc.** **(Minato, TKY)**	7-(4,6-Di-tert-butyl-pyrimidin-2-yl)-3-(4-tri-fluoromethoxy-phenyl)-5,6,7,8-tetrahydro-[1,2,4]triazolo[4,3-a]pyrazine derivative, IC_50_ = 19 nM *in vitro* assay, Significant reduction of TAG excursion in a mice olive oil oral gavage model, >300-fold selective over MGAT3, and >1000-fold selective over DGAT2,	[68]

The first patent describing MGAT2 inhibitors was published in 2010 (WO2010/095767, Banyu Pharmaceutical Co. Ltd., Tokyo, Japan), where pyrimidine-4(3H)-one derivatives were identified. The inhibitory activity (IC_50_ = 56 nM) of these compounds was examined in a radioactive-labeling assay with membrane fractions prepared from human MGAT2-expressing yeast. Bicyclic pyrimidine derivatives (WO2012/091010, Dainippon Sumitomo Pharma, Japan) and *N*-containing heterocyclic derivatives (WO2012/124744, Taisho Pharmaceutical Co. Ltd., Tokyo, Japan) were next identified. Both of these chemotypes showed improved inhibitory effects against MGAT2 in a radio-labeling enzyme assay using the membrane fractions prepared from human MGAT2-expressing Sf-9 cells; IC_50_ = 4.1 nM and IC_50_ = 2 nM, respectively. A non-radioactive fluorescence assay was later developed for the *N*-containing heterocyclic derivatives using a 7-diethylamino-3-(4-malimidophenyl)-4-methylcoumarin (CPM) fluorescent substrate assay that detected the release of CoASH from MGAT2 using a membrane fraction prepared from human MGAT2-expressing Sf-9 cells.

Aryl dihydropyridinones and piperidinones derivatives (WO2013/082345, WO2014193884 Bristol-Myers Squibb Co., New York, NY, USA) inhibited MGAT2 with an IC_50_ = 14 nM in a radioactive-labeling enzyme reaction assay using human MGAT2-expressing Sf-9 cell membrane fraction. Using a stable radioactive-labeled substrate and high-resolution LC/MS with human MGAT2-expressing STC-1 cell based assay, these chemotypes demonstrated IC_50_ = 4 nM and selectivity over MGAT3 (>1000-fold), an acyl-CoA wax alcohol acyltransferase 2 (>1000-fold), and DGAT1 (>1000-fold) [65]. Phenyl methanesulfonamide derivatives that were developed by Eli Lilly (WO2013112323) had excellent MGAT2 inhibitory effects (IC_50_ ~12 nM) in a radioactive-labeling enzyme reaction assay using a human MGAT2-expressing Sf-9 cell membrane fraction. In ahumanMGAT2-expressing Caco2 cell based LC-MS assay, the chemotype inhibited MGAT2 with IC_50_ = 17.7 nM. The benzyl sulfonamide derivatives (WO2014074365, Eli Lilly, USA) showed an improved MGAT2 inhibitory effect over the phenyl methanesulfonamide derivatives with IC_50_ = 2.28 nM in a radioactive-labeling enzyme reaction assay using a human MGAT2-expressing Sf-9 cell membrane fraction. In human MGAT2-expressing Caco2 cell based LC-MS assay, the chemotype inhibited MGAT2 with IC_50_ = 3.8 nM. In a dog oil bolus model, the compounds were dosed at 10mg/kg and reduced TAG circulation by more than 70%.

Morpholynyl derivatives (US20150005305, Eli Lilly, USA) showed MGAT2 inhibitory activity with an IC_50_ = 12 nM in a radioactive-labeling enzyme reaction assay using a human MGAT2-expressing Sf-9 cell membrane fraction. In human MGAT2-expressing Caco2 cell based LC-MS assay, the chemotype inhibited MGAT2 with an IC_50_ = 16 nM. Finally, in a dog oil bolus model where compounds were dosed at 30mg/kg and 75mg/kg, TAG circulation was reduced by 43% and 64%, respectively.

The singleton 3-ethyl-3-methyl-2,5-dioxo-*N*-phenyl-2,3,4,5-tetra hydro-1H-1,4-benzodiazepine-7-sulphonamide 1 [66] showed MGAT2 inhibitory activity using a Rapidfire LCMS^®^ assay, giving an IC_50_ = 1.6 nM.The singleton had > 10,000-fold selectivity over the acyl-CoA/wax alcohol acyltransferases, AWAT1/AWAT2, and DGAT1. It had variable selectivity over MGAT3 and no selectivity towards MGAT1, and achieved a high bio-availability, while reducing circulating TAG after corn oil oral gavage by 68% in mice.

*N*-phenylindoline-5-sulfonamide derivatives [67] achieved an IC_50_ = 3.4 nM towards MGAT2 and >30,000-fold selectivity over MGAT3, DGAT1, DGAT2, and ACAT1. They are highly bio-available and effectively suppress post-olive-oil-loaded TG excursion after oral gavage in C57BL/6J mice.

JTP-103237, a 7-(4,6-Di-tert-butyl-pyrimidin-2-yl)-3-(4-tri-fluoromethoxy-phenyl)-5,6,7,8-tetrahydro-[1,2,4]triazolo[4,3-a]pyrazine derivative, potently inhibited MGAT2 activity in a rat intestinal S9 fraction and membrane fractions of MGAT2-expressing COS-7/Sf9 cells using radioactive-labeling assay [68]. JTP-103237 has an IC_50_ = 19 nM which is >300-fold selective over MGAT3 and >1000-fold selective over DGAT2. Plasma PYY level, but not GLP-1 was increased after administrating JTP-103237 in rats. As PYY is a satiety factor, this may explain the decreased cumulative food intake in rats fed a 35% fat diet and JTP-103237 when compared to control group. JTP-103237 not only prevented high-fat diet induced metabolic syndromes, it also caused a significant decrease in body weight, plasma glucose levels, and food intake in obese BDF1 mice fed with high-fat diet. Interestingly, the decreased glucose level seen and decreased food intake brought about by JTP-103237 is more significant than the marketed anti-obesity drug orlistat, a pancreatic lipase inhibitor. In addition, JTP-103237 also reduced fat weight and hepatic TAG content.

## 4. Conclusion

**Figure 2:**
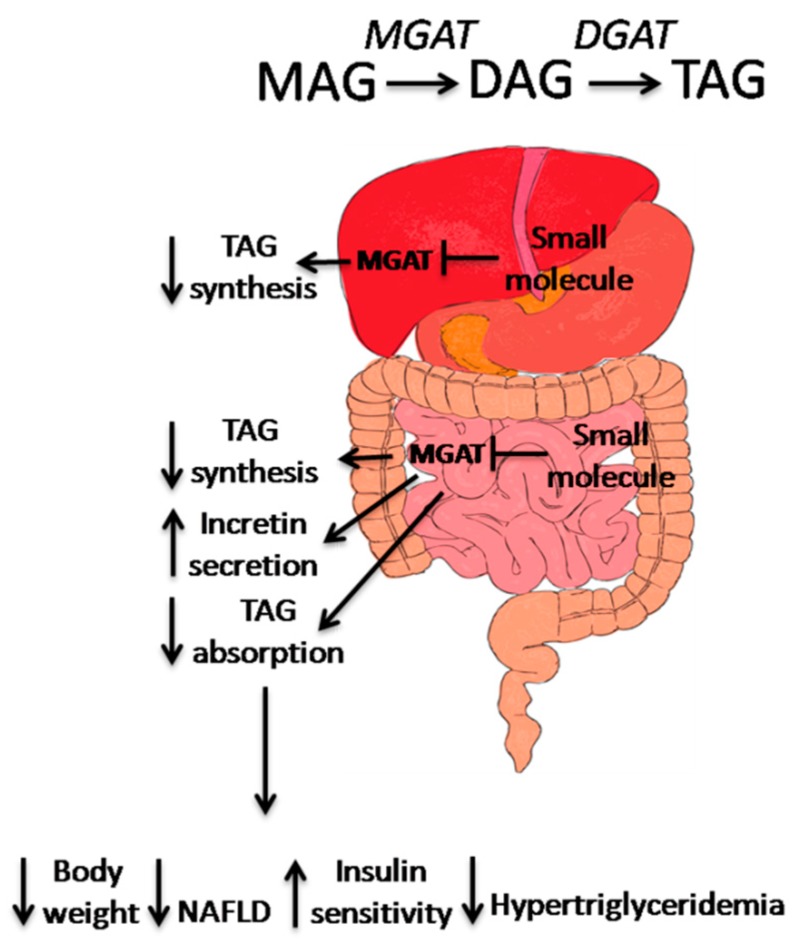
The mode of action of MGAT2 inhibitors in the treatment of metabolic disorders. In mouse liver, inhibition of MGAT1 has shown to improve insulin sensitivity as well as hepatic steatosis. In human liver, MGAT2/MGAT3 expression is correlated with progression of NAFLD, in the small intestine, MGAT2 inhibition results in changes in TAG absorption and synthesis, as well as incretin secretion. These actions contribute to the weight loss, improvement of insulin sensitivity and hypertriglyceridemia, and prevention of NAFLD progression.

Excessive TAG synthesis in the intestine due to dietary fat absorption followed by increased accumulation of TAG in the liver and adipose plays an integral role in the progression of metabolic disorders including obesity, insulin resistance, T2D, and fatty liver disease (Figure 2). 

Blocking dietary TAG absorption and resynthesis in the intestinal lumen has been seen as a viable pathway in which to design small molecules targeting obesity and metabolic syndrome; inhibitors to the intestinal lipase (e.g., orlistat) are currently one of the few medicines available on the market, and inhibitors to DGAT1 are currently in clinical trials. However, both types of inhibitors exhibit unwanted gastrointestinal side effects [63,69]. Inactivation of MGAT2 by genetic manipulation and pharmacological intervention supports the idea that targeting the MAG pathway as a therapeutic for metabolic syndrome is a viable option for inhibiting intestinal TAG synthesis. It has already been shown that inhibiting intestinal MGATs, especially MGAT2, results in dynamic changes in TAG and cholesterol absorption, which leads to the changes in systemic energy balance and gut incretin release. It is also possible that inhibition of the MGAT2 isozyme in the liver will improve steatosis by attenuating fat accumulation and insulin resistance. In adipose, MGAT2 inhibition may reduce fat biosynthesis and improve glucose uptake. Based on all the data accumulated thus far, the combined effects of MGAT2 inactivation in various tissues will have beneficial effects in reducing body weight, improving insulin resistance, decreasing hyperlipidemia, and attenuating hepatic steatosis.

## Abbreviations

NAFLD: nonalcoholic fatty liver disease;
TAG: triacylglycerol;
MGAT2: acyl-coA: monoacylglycerolacyltransferase 2;
MAG: monoacylglycerol;
DAG: diacylglycerol;
FA: fatty acid;
G3P: sn-glycerol-3-phosphate;
DGAT: acyl-coA: diacylglycerolacyltransferase;
GPAT: glycerol-3-phosphate acyltransferase;
LPA: lysophosphatidic acid;
AGPAT: acylglycerol-3-phosphate acyltransferase;
Pa: phosphatidic acid;
Lipin/PAP: lipins/PA phsphohydrolases;
ER: endoplasmic reticulum;
DHAP: dihydroxyacetone phosphate;
VLDL: very low density lipoprotein;
GLP-1: glucagon-like peptide-1;
RYBG: Reux-en-Y gastric bypass;
DIO: diet-induced obese;
PPAR: peroxisome proliferator-activated receptor;
ASO: antisense oligonucleotides;
HTF-C diet: high trans FAs, fructose and cholesterol diet;
NASH: nonalcoholic steatohepatitis.
